# Interferon Crevicular Fluid Profile and Correlation with Periodontal Disease and Wound Healing: A Systemic Review of Recent Data

**DOI:** 10.3390/ijms19071908

**Published:** 2018-06-29

**Authors:** Luca Fiorillo, Gabriele Cervino, Alan Scott Herford, Floriana Lauritano, Cesare D’Amico, Roberto Lo Giudice, Luigi Laino, Giuseppe Troiano, Salvatore Crimi, Marco Cicciù

**Affiliations:** 1 Department of Biomedical and Dental Sciences and Morphological and Functional Imaging, Messina University, 98122 Messina, Italy; lucafiorillo@live.it (L.F.); gcervino@unime.it (G.C.); flauritano@unime.it (F.L.); cesaredamico89@gmail.com (C.D.); roberto.logiudice@hotmail.it (R.L.G.); 2 Department of Maxillofacial Surgery, Loma Linda University, Loma Linda, CA 92354, USA; aherford@llu.edu; 3 Multidisciplinary Department of Medical-Surgical and Odontostomatological Specialties, University of Campania, Luigi Vanvitelli, 80121 Naples, Italy; luigi.lainoi@unicampania.it; 4 Clinical and Experimental Medicine, University of Foggia, 71121 Foggia, Italy; giuseppe.troiano@unifg.it; 5 Department of Surgical and Biomedical Sciences, Catania University, 95124 Catania, Italy; torecrimi@gmail.com

**Keywords:** periodontitis, interferon, autoimmune, anti-interferon

## Abstract

The purpose of the present study was to see if there is a correlation between the effect of interferons in crevicular fluid and periodontitis, evaluating literature. Interferon gamma (IFN-γ) is an immunoregulatory cytokine that, when activated by its receptor, plays an important role in the activation of inflammatory processes, which are the basis of periodontal disease. Stem cells in the periodontal ligament, like stem cells from other tissues, have immunomodulatory capacity and are regulated by some cytokines such as interferon-γ (IFN-γ). The study searched MEDLINE databases from 2008 to 2018. Clinical human in vitro and in vivo studies had reported a correlation between interferon and periodontitis. The initial search obtained 359 citations. After screening and determination of eligibility, nine articles were included in the review. Significant (*p* < 0.05) increases in *IFN-γ* gene expression were observed in some studies in the chronic periodontitis group. In some cases it was suggested that molecular mechanisms underlie the possible roles of IFN-γ in the inhibition of osteoclastogenesis. Neopterin belongs to the chemical group known as pteridines. It is synthesised by human macrophages upon stimulation with the interferon-gamma. Neopterin concentrations in body fluids are high in the case of infections, immune diseases or graft rejection. In the chronic periodontitis group, this marker is significantly higher. These studies underlined the clinical evidence between interferons in the crevicular fluid and inflammatory response of periodontitis. However, there is a lack of scientific evidence that could lead the clinician to an interferon-modulated therapy because of periodontitis.

## 1. Introduction

Periodontitis is associated with an increased risk of diseases such as atherosclerotic vascular disease and type 2 diabetes. This could be due to the release of bacterial products and inflammatory mediators from periodontal pockets into the bloodstream, resulting in low-grade systemic inflammation. Proinflammatory (interferon (IFN-γ)) markers have been identified in serum or gingival crevicular fluid. The purpose of the present study was to see if there is a correlation between the effect of interferons in crevicular fluid and periodontitis by evaluating the literature. Interferon gamma (IFN-γ) is an immune regulatory cytokine that works through its receptor and plays an important role in the progression of inflammation. In chronic periodontitis, the stem cells of the periodontal ligament, like the stem cells of other tissues, possess immunomodulatory properties, which are regulated by different cytokines, such as by interferon-γ (IFN-γ). Variations of this value in the crevicular fluid may or may not indicate a pathological state; it is therefore important to evaluate the results of the different studies to provide an important means of diagnosis for a state of oral inflammation such as the periodontics, which may have systemic implications [1]. Interferon-gamma plays a role in other major diseases, concerning other body systems, too. IFN-γ is a homodimeric protein that belongs to the type II cytokines. Its receptor is made up of two polypeptide chains, belonging to the same family as IFN-γ and called IFNGR1 and IFNGR2. The binding of IFN-γ with the two chains induces their dimerization, which causes an association between the JAK1 and JAK2 kinases, activating the JAK/STAT signalling pathway. This signalling pathway leads to the activation of the transcription factor STAT1, which induces the transcription of several genes, including IFN-γ itself, which activates macrophages to kill the phagocytosed microorganisms together with the CD40L-CD40 binding; furthermore, IFN gamma promotes differentiation in Th1 by activating the transcription factor T-bet and inhibits that towards Th2. Interferon gamma performs multiple functions in different cells of the organism:

- In macrophages: Causes classical activation along with Toll-like receptor signals. In these cells it activates numerous transcription factors such as STAT1, NF-κB and AP-1 that induce the transcription of different enzymes involved in phagocytosis, such as phagocytic oxidase, inducible nitric oxide synthase and other lysosomal enzymes.
- In B lymphocytes: Promotes class exchange between the various Ig subclasses.
- In TH1 lymphocytes: Stimulates the CD4 T lymphocytes to differentiate into TH1 lymphocytes, inhibiting TH2 and TH17.
- In MHC: Acts on MHC molecules, inducing the expression of proteins that improve the binding with the peptide and the consequent presentation [2].

IFN-γ and different cytokines, i.e., interleukin (IL)-2, IL-4, IL-6, are present in advanced and generalized chronic periodontitis. Some subunits of IFN-γ like IFN-γ-R1 were detected at an early stage during the active phase of the disease, but the expression of positive cells remained unaltered during the remaining period of study. IFN-γ plays a role in bone resorption, too, and its presence seems to be significant in healing after periodontal disease therapy. Increases in bone volume and trabecular bone number and decreases in trabecular separation revealed the antiosteoclastic activity of IFN-γ [3,4,5].

## 2. Results

As shown in the studies analysed, broadly developed in Table 1, there is no evidence that can lead to the demonstration of a correlation between percentages of IFNγ in the crevicular fluid and periodontitis. Studies where this phenomenon is evident do not have a sufficient sample size. In other works, the results were actually the opposite, with a correlation between oral health status and an increased percentage of IFNγ. The values of significance that we obtained are only demonstrated by Shaddox et al., with higher percentages of IFNγ (*p* < 0.001) [6] in the sites taken into consideration compared to healthy sites. Still, similar values were obtained by Thunell (*p* < 0.0001) [7] and Marazumi (*p* < 0.001) [8]. Finally, Zhang et al. found a correlation between the transcriptional levels of IFNγ and the periodontitis with values of *p* = 0.04 [9].

### 2.1. Study Selection

**Figure 1:**
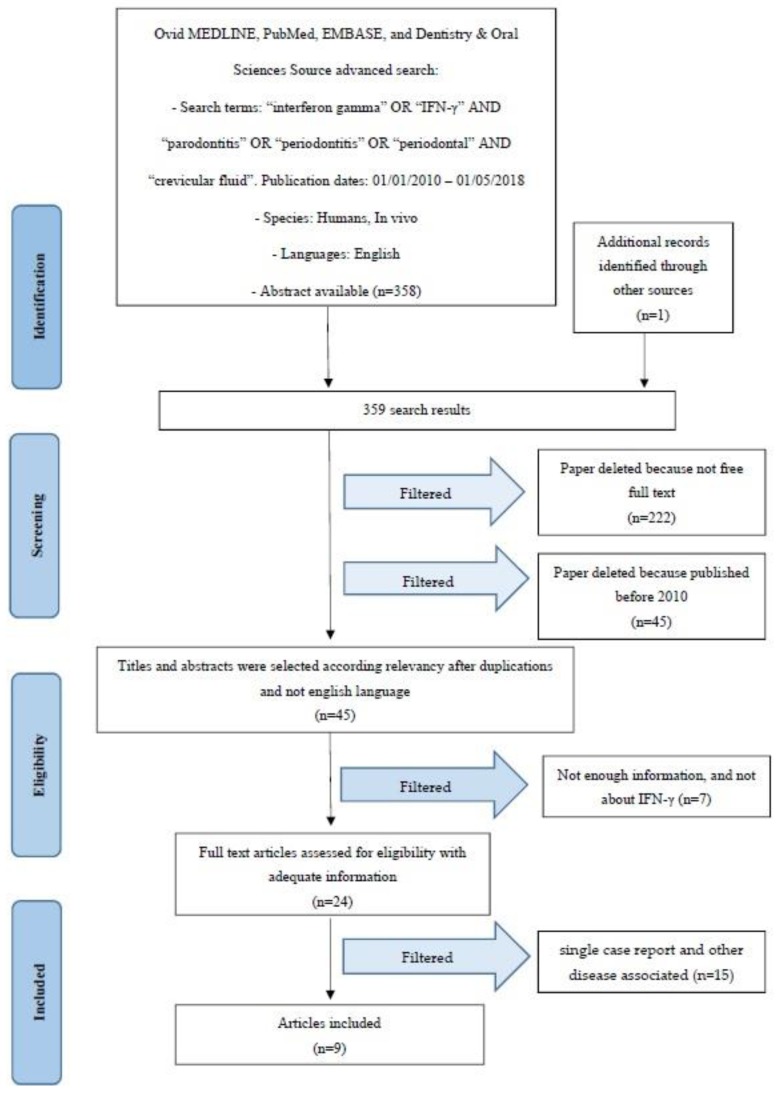
PRISMA flow diagram.

Article review and data extraction were performed according to the PRISMA flow diagram (Figure 1). The initial electronic and hand search retrieved 358 citations and one more paper from Dentistry, Pubmed Medline and Oral Sciences Source with a total of 359 selected papers. After the titles and abstracts were reviewed, 222 non-full-text articles were filtered out, 45 were removed because they were published before 2010, and seven were deselected for not having enough information regarding the selected topic. Finally, nine articles were included in this review.

### 2.2. Study Characteristics

After the study selection, a new division related to the kind of inflammatory mediators was performed, taking IFNγ as the primary consideration and evaluating the method used in the different studies analysed.

IFNγ evaluation: six studies [6,7,9,10,11,12].

After periodontal treatment measurement: five studies [7,8,10,11,12].

Inflammatory mediators type of investigation: Luminex^®^: four studies [6,9,10,12]; Millipex^®^: one study [6]; PCA-RT^®^: one study [13].

### 2.3. Risk of Bias within Studies

Summarizing the risk of bias for each study, most of the studies were classified as having unclear risk [14]. More studies were considered as having a low risk of bias [9,11], whereas another one was classified as having moderate risk [7,13], and three studies were attributed a high risk of bias [6,8,10,12,14].

### 2.4. Risk of Bias across Studies

There were several limitations to the current review. It includes only English-language studies, which could introduce a certain risk of bias. The included studies have a relatively short follow-up and a small number of patients have been evaluated. There are different degrees of heterogeneity in the design of each work taken into consideration, case selection, and treatment provided among studies. The absence of control groups was an additional limitation. There was also too high a heterogeneity of the selected studies to compare the impact of different periodontal therapy for the final treatment outcome.

## 3. Discussion

**Figure 2:**
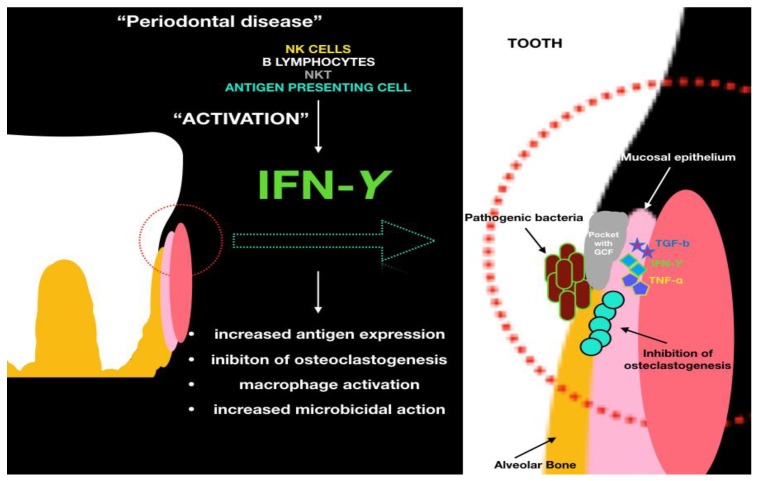
The role of IFN-γ and the cellular mechanisms involved in the wound healing process.

Nowadays periodontitis is a disease that involves many people in the world, is widespread in all countries and that affects every gender and ethnicity. Trying to prevent it through treatment and avoidance certainly improves the quality of life of individuals and ensures that teeth are kept in the mouth longer. Already in the 1960s the possibility of screening was being evaluated so as to prevent and resolve the disease before any irreversible damage, such as tooth loss [15]. The phenomena involved in this pathology are multiple, just like its aetiology. In this study we try to understand the role of the biological mediators in periodontitis, in particular IFN-γ (Figure 2).

Cytokine proteins may have important roles during different human physiological and pathological processes. For example, the IL-1β concentration changes in GCF suggest these cytokines as a predictable marker of gingival inflammation in chronic periodontitis patients [16,17]. It results from studies in which periodontal disease is related to other general disease as cardiovascolar ones [18]. Evidence of an association between periodontitis and atherosclerotic vascular disease, including stroke, myocardial infarction, peripheral vascular disease, abdominal aortic aneurysm, coronary heart disease and cardiovascular death, comes from more than 50 prospective cohort and case control studies undertaken during the last 25 years. More recent analyses from large cohort studies suggest that new-onset as well as prevalent periodontitis is associated with increased coronary heart disease risk, and there is a graded association between tooth loss and stroke, cardiovascular death, and all-cause mortality in patients with stable coronary artery disease [19]. There is not much information regarding the correlation between oral health and patients with neurodegenerative diseases. However, available reports demonstrate that many oral health problems, as evidenced by poor oral hygiene, higher caries prevalence, periodontal diseases, and lower saliva flow rates, were found in subjects with neurodegenerative disease when compared with healthy individuals of the same age. The international community is currently debating how oral health status impacts a patient’s quality of life [20,21].

In some cases, however, patients are affected by complex primary pathologies that make treatment difficult, such as decayed, missing, and filled teeth (DMFT) and periodontal disease in BRONJ (Biphosphonated Related Ostoenecrosis of the Jaws) patients, which is connected with the occurrence of jawbone necrosis [22]. The presence of periodontitis involves chronic oral inflammation, and with this the presence of a bacterial inflammatory site.

The implant prosthetic therapy is increasingly requested and increasingly used nowadays, especially thanks to new regenerative surgical techniques using next-generation materials and marine animal derivation [23]. New materials and implant mechanics allow for an adequate discharge of mechanical forces, ensuring the predictability of the results [24]. Unfortunately, even implants may be affected by the inflammatory process, and in the case of periodontal patients there may be correlations; this study is even more valuable in that sense. By understanding the biological mechanisms of this pathology it may be possible to block it or slow it down in both teeth and implants, while also considering surgical implants or proper reconstruction for the long-term maintenance of the patient’s health. Most patients do not consider the importance of management due to implant therapy. In most cases these patients are not informed about the risk of implant loss and the factors that influence the predictability of the therapy. For example, a remission of the destructive inflammatory lesion on experimentally induced peri-implantitis tissues was seen only in some sites following ligature removal, but in the majority of sites additional loss of supporting bone occurred [25,26,27].

Lomba et al. demonstrated that immunohistochemistry did not show significant differences between shallow and deep sites for IL-1-β as well as for IFN-γ, IL-6 and IL-17. IFN-γ was observed in all cell groups for the two groups analysed. Thus, according to the methodology adopted, it was not possible to determine significant differences in immunohistochemical markers between shallow and deep sites. When this agreement percentage was evaluated separately between shallow and deep sites, we observed a higher percentage of agreement in the group of deep sites for all four markers analysed, reaching 100% for IL-1-β, 81% for IL-6, 75% for IL-17, and 63% for IFN-γ. This result could be related to the increased epithelial permeability in deep sites, as that increases with the progression of inflammation [9].

Arjunkumar et al., in a comparative analysis, demonstrated that neopterin levels in GCF are positively associated with periodontal disease, which may prove a useful tool in monitoring its progression. The neopterin level was significantly higher than in the control group [14].

Shaddox et al. noted that *P. gingivalis* LPS induction of IFNγ was significantly decreased (*p* < 0.05). Similarly, *E. coli* LPS induces a response by INFγ that was also reduced post-treatment. The inflammatory response caused by the LPS can be modulated with therapy. The presence of increased clinical parameters suggests that this phenotype can be influenced by genetic traits that play a fundamental role in the evolution of the disease and its recurrence [10].

In 2013, Fu et al. found that removal of dental plaque resulted in an improvement of all clinical parameters, while GCF IFN-γ and IL-10 levels remained unchanged [11].

Results were similar for de Lima Oliveira et al. with Luminex^®^ investigation and periodontal therapy, where periodontal therapy improved GCF cytokine profiles by lowering IL-1β and increasing IL-10 levels. The reduction in GCF GM-CSF after therapy implicates this cytokine in the pathogenesis of GAgP. There was no difference between therapies in terms of changes in GCF cytokines and IFN-γ levels [12].

Shaddox et al. found that nine mediators, including INFγ, were elevated in LAP diseased sites as compared with healthy sites, while MCP1, IL4, and IL8 were elevated in healthy sites [6].

Zhang et al. observed that a hypomethylation profile within IFNG promoter region is related to an increase of IFNG transcription present in the chronic periodontitis biopsies, while such an increase of IFNG in experimentally induced gingivitis seems independent of promoter methylation alteration. The findings from this study indicate the potential role of local epigenetic effects that result in regional modifications of the tissue DNA methylation status in the pathogenesis of chronic inflammatory periodontal disease. Because the DNA methylation status can be modified by certain drugs, the possibility of reversing epigenetic modifications may have profound effects on periodontal treatment responses [13].

Thunell et al. demonstrated that periodontal therapy effectively reduces pro-inflammatory cytokines and chemokines, including less well-described mediators that may be important in the initiation and progression of periodontitis, but the problem with this study is the small sample size (only six) [7].

Morozumi et al. observed that scaling and root planing resulted in clinical and microbiological improvement, but produced a moderate systemic acute-phase response and elevated inflammatory mediators one day post-treatment, including a significant elevation in body temperature and a remarkable increase in interferon-γ (*p* < 0.001) [8].

### Limitations

Although a comprehensive investigation of the effects of surgical therapies has been performed, there were some limitations to this systematic review. Our findings could not provide the ideal therapy for periodontitis or the ideal diagnostic method. Moreover, the reviewing only English papers it could be considered a new potential language bias.

## 4. Material and Methods

### 4.1. Protocol and Registration

This review is registered at PROSPERO with ID number 98536. CRD York and Prospero website offer an international database for registering revision paper in the health care field. The present work has been recorded in the website for having permanent record.

### 4.2. Focus Question

The following focus question was developed according to the population, intervention, comparison, and outcome (PICO) study design: 

Is there a correlation between the levels of interferon gamma in the crevicular fluid and periodontitis?

As an alternative focused question, can the interferon gamma represent a diagnostic and preventive means for periodontitis?

### 4.3. Information Sources

The search includes an examination of the electronic databases in addition to a manual search of the literature. A search of four electronic databases, including Ovid MEDLINE, PubMed, Dentistry, and Oral Sciences Source, for relevant studies published in the English language from January 2010 to March 2018 was carried out. The search was limited to English-language articles. The references of the articles obtained were also evaluated to increase the relevance of the work and improve the sensitivity of the search.

### 4.4. Searches

The keywords used in the search of the selected electronic databases included the following: “interferon gamma” OR “IFN-γ” AND “parodontitis” OR “periodontitis” OR “periodontal” AND “crevicular fluid”.

The choice of keywords was intended to collect and record as much relevant data as possible without relying on electronic means alone to refine the search results.

### 4.5. Selection of Studies

Two independent reviewers analysed the obtaining papers independently in order to select inclusion and exclusion criteria as follows. For the stage of reviewing full-text articles, a complete independent dual revision was performed.

### 4.6. Types of Manuscripts Selected

The review included studies on humans and animals published in the English language. Letters, editorials, case reports, and PhD theses were excluded.

### 4.7. Types of Studies

The review included all human prospective and retrospective follow-up studies and clinical trials, cohort studies, case–control studies, and case series studies, animal studies and literature review published between January 2010 and March 2018, on interferon gamma and periodontitis correlation.

### 4.8. Disease Definition

Periodontitis, also called periodontitis and parodontopathy, is an inflammation of the periodontal tissues, which causes a loss of attachment of the teeth with respect to the alveolus, with consequent formation of periodontal pockets, dental mobility, gingival bleeding, abscesses and suppurations, up to the loss of one or more teeth. This process is reversible if it is diagnosed in its early stages and treated. As the disease progresses, measured primarily as a progression of periodontal attachment loss, the possibility of recovery becomes more difficult and requires more complex treatments such as regenerative bone therapy. Recovery in these cases is generally partial. In 2015 about 538 million people result affected by periodontal disease. Epidemiological studies performed on US patients recorded how about the 70% is about over the age of 65 years old and males are more involved in the periodontal disease despite the females patients [28,29,30].

### 4.9. Inclusion and Exclusion Criteria

The full text of all studies of possible relevance was obtained for assessment against the following inclusion criteria:

- Correlation between interferon gamma levels and periodontitis.
- Periodontitis and biomarker levels in the crevicular fluid.
- Periodontal therapy and variation of inflammatory mediators levels in the crevicular fluid.
- Correlation between levels of interferon oral ranges and diseases in other districts.
- All human prospective or retrospective follow-up studies and clinical trials, cohort studies, case–control studies, and case series studies with a six-month follow-up.
- Animal or in vitro studies.

The applied exclusion criteria for studies were as follows:

- Studies involving patients with specific diseases, immunologic disorders, uncontrolled diabetes mellitus, osteoporosis, or other implant risk-related systemic conditions.
- Not enough information regarding the selected topic.
- Articles published prior to 1 January 2010.
- No access to the title and abstract in English language.

### 4.10. Sequential Search Strategy

After the first literature analysis, all article titles were screened to exclude irrelevant publications, case reports and non-English-language publications. Then, studies were deselected based on data obtained from screening the abstracts. The final stage of screening involved reading the full text to confirm each study’s eligibility based on the inclusion and exclusion criteria.

### 4.11. Data Extraction

The data were obtained by evaluating the aims of the work and the themes of the present review.

### 4.12. Data Collection

Data were collected from the included articles and arranged in the following fields:

- “Author (Year)”—Revealed the author and year of publication.
- “Type of study”.
- “Sample size”—Described the number of patients, animals or models examined.
- “Inflammatory mediators investigated”—Described types of inflammatory mediators evaluated in studies.
- “IFN-gamma”—Interferon gamma levels evaluated or not.
- “Statistics”—Described the presence of significant or nonsignificant mediator levels.

### 4.13. Risk of Bias Assessment

The risk of bias was limited due to the use of multiple authors during the phase of information extraction from the studies taken into consideration. The researches selected in the present revisions underwent the Cochrane Collaboration’s two part tool for the risk of bias evaluation [31,32].

The following possible sources of bias were addressed: random sequence generation (selection bias); allocation concealment (selection bias); incomplete outcome data (attrition bias); and selective reporting (reporting bias). For each article taken into consideration in the review, a judgment of the author has been added (Table 1). The risk has therefore been reduced by evaluating and respecting the parameters of Higgins et al. [32]. The levels of bias were classified as follows: low, if all the criteria were met; moderate, when only one criterion was missing; high, if two or more criteria were missing; and unclear, if too few details were available to make a risk assessment.

## 5. Conclusions

The purpose of this work was to shed light on the most important inflammatory mediators taking part in periodontitis. The main focus was on INF-γ and its variations in crevicular fluid in the case of healthy patients, periodontal patients and post-treatment patients. The data are not sufficient to suggest an effective therapy based on the involvement of inflammatory mediators. The data are still discordant: some authors show an increase of this value in case of inflammation, while others notice a decrease, and therefore the presence of high percentages in healthy sites. Although we do not have conclusive results, this work is an important starting point for assessing the importance of the periodontal disease and its correlation with the interferon Y. It is necessary to consider that patients are increasingly turning to implant rehabilitation, as mentioned earlier. It is not yet clear, due to researchers’ discordant opinions, whether interferon is more prevalent in healthy tissues or in tissues affected by disease. Periodontitis may cause implant rehabilitation problems and a clinician’s multi-specialist and periodontal approach is required if implant therapy is to be avoided.

## Figures and Tables

**Table 1:** Summary of studies involved in the present revision.

Reference	Authors	Type of Investigation	No. of Sample (Control)	Mediators Evaluated	IFN-γ	Results	Statistics
[6]	Shaddox et al.	Milliplex^®^, Luminex^®^	34 (+10 controls)	TNF-α, IFN-γ, IL-1β, IL-2, IL-10, IL-12p40, GMCSF, M1P1a	Yes	Diseased sites from LAP participants presented higher levels of IFN-γ when compared with levels in their own healthy sites.	*p* < 0.001
[7]	Thunell et al.	Periodontal treatment	6	IFN-γ, IL-1α, IL-1β, IL-2, IL-13, IL-3, IL-4, IL-6, IL-12p40, GMCSF, TNF-α	Yes	IFN-γ is significantly higher in sites	*p* < 0.0001
[13]	Zhang et al.	PCA-RT	12 (+47 controls)	mRNA expressions of IFN-γ	No	The transcription levels of IFN-γ was increased and significantly higher in the periodontitis biopsy samples	*p* = 0.04
[9]	Lomba et al.	Luminex^®^	22	IFN-γ, IFN-1β, interleukin-6, interleukin-17α, interleukin-17F	Yes	There are differences between mediators in swallow and deep sites.	IFN-γ *p* = 0.233
[10]	Shaddox et al.	Luminex^®^, periodontal treatment	59	IFN-γ, IL10, IL12p40, IL1β, IL6, MCP1, GM-CSF, IP10, TNFα	Yes	Periodontal treatment resulted in significant improvement in most clinical parameters and LPS induced inflammatory response.	*E. Coli*: *p* = 0.14; *Porphyromonas gingivalis*: *p* = 0.001
[11]	Fu et al.	Periodontal treatment	148 (+132 control)	IFN-γ, IL10, IL17	Yes	Experimental studies have demonstrated that IFN-γ positive cells * as Th1 cells play a prominent role in mediating periodontal tissue destruction	No significant differences after therapy
[12]	Oliveira et al.	Luminex^®^, periodontal treatment	895	IFN-γ, IL-10, IL-1β, IL-2, IL-6, TNF-α	Yes	No effect of periodontal therapy on GVF levels of IFN-γ	*p* = 0.4–0.7
[8]	Marazumi et al.	Periodontal treatment	39	IFN-γ, IL-4, IL-5, IL-6, IL-12p70, TNF-α	Yes	After treatment, significant levels of IFN-γ were present	*p* < 0.001
[14]	Arjunkunan et al.	/	30	Neopterin	No	Upon stimulation by IFN-γ, neopterin is released, but there is no significant difference between groups	*p* > 0.05
